# ImmuniT Platform for Improved Neoantigen Prediction in Lung Cancer

**DOI:** 10.3390/vaccines13090921

**Published:** 2025-08-29

**Authors:** Stephanie J. Hachey, Alexander G. Forsythe, Hari B. Keshava, Christopher C. W. Hughes

**Affiliations:** 1 Molecular Biology and Biochemistry, University of California, Irvine, CA 92697, USA; cchughes@uci.edu; 2 Individualized Interdisciplinary Studies, Simon Fraser University, Burnaby, BC V5A 1S6, Canada; 3 Division of Thoracic Surgery, Department of Surgery, University of California, Irvine, CA 92697, USA; keshavah@hs.uci.edu

**Keywords:** lung carcinoma, neoantigen, immunotherapy, personalized therapy

## Abstract

Background: Lung cancer remains the leading cause of cancer-related mortality, with many patients responding poorly to immunotherapy due to limited tumor recognition. Neoantigen-based strategies offer a promising solution, but current discovery methods often miss key targets, particularly those with low or heterogeneous expression. To address this, we developed ImmuniT, a three-phase platform for enhanced neoantigen discovery and validation. Methods: Under an IRB-approved protocol, patients with lung cancer consented to tumor collection for ex vivo processing to modulate antigen expression. Autologous T cells from matched blood were co-cultured with treated cancer cells to expand tumor-reactive populations. The nextneopi pipeline integrated mutational, transcriptomic, and HLA data to predict candidate neoantigens, which were validated using MHCepitope tetramer staining. Results: In five patient samples, ImmuniT identified a broader spectrum of neoantigens and induced stronger T cell activation in vitro compared to conventional approaches. Notably, in one case, two neoantigens missed by standard methods were confirmed to elicit tumor-specific T cell responses in both the tumor-infiltrating and peripheral compartments. Conclusions: These findings highlight ImmuniT’s potential to expand the repertoire of actionable tumor antigens and improve personalized immunotherapy strategies, particularly for patients with limited response to existing treatments.

## 1. Introduction

Lung cancer remains the leading cause of cancer-related deaths in the US and globally, with 85% of patients presenting with advanced, treatment-resistant disease [1]. Non-small cell lung cancer (NSCLC), the predominant subtype, is characterized by a high tumor mutational burden (TMB), which has been associated with improved responses to immunotherapy [2]. However, despite the success of immune checkpoint inhibitors (ICIs), only 20–25% of patients derive a significant clinical benefit, largely due to insufficient immune recognition of tumors [3,4]. NSCLC’s heterogeneity and genetic instability drive both disease progression and therapeutic resistance, yet these same factors provide an opportunity to develop patient-specific neoantigen-targeted therapies.

Neoantigens, which arise from tumor-specific somatic mutations, play a critical role in anti-cancer immune responses by eliciting tumor-reactive T cell activity [5]. Early clinical trials have demonstrated that immunotherapy can induce neoantigen-specific immune responses in NSCLC patients [6,7,8,9]. However, a major challenge remains: while NSCLC tumors harbor an average of 75 neoantigens, these are often heterogeneously expressed within the tumor, limiting their therapeutic potential [10,11]. Current neoantigen discovery approaches struggle to identify highly immunogenic targets, particularly those expressed at low levels or restricted to rare tumor cell populations, such as cancer stem cells [12]. This limitation allows the cancer to evade immune attack and develop resistance to therapy.

Despite advances in sequencing, bioinformatics, and mass spectrometry, the identification of tumor-specific neoantigens remains challenging [13,14]. Shared antigens across different cancers have shown limited efficacy, shifting the focus to patient-specific tumor antigens [4]. While neoantigen-reactive T cell therapy has demonstrated promising results, its full therapeutic potential hinges on the accurate identification of highly immunogenic targets. To address this, we have developed the ImmuniT platform, which surpasses conventional methods by detecting both abundant and low-level neoantigens. This platform amplifies patient-specific neoantigens directly from primary tumor samples, leading to improved prediction of neoantigens for personalized immunotherapies. The ImmuniT platform is a complete pipeline encompassing both the induction of neoantigen expression in tumor cells via a defined cocktail of drugs and growth factors, and a subsequent neoantigen discovery and T cell priming workflow.

## 2. Materials and Methods

### 2.1. Tissue and Blood Collection

Surgically resected NSCLC tissue and matched blood samples were collected from 20 patients under an IRB-approved protocol at the University of California, Irvine. All experimental procedures were conducted in accordance with institutional guidelines and regulations, with informed consent obtained from all participants or their legal guardians. Adjacent normal lung tissue, resected beyond tumor margins to ensure complete disease removal, was also included in the study. Blood samples were collected in 7.5 mL CPT Mononuclear Cell Preparation tubes containing sodium heparin and Ficoll. Surgical resections were collected in a conical tube containing transport media and transported on ice within 2 h of collection. The transport media consists of DMEM (4.5 g/L glucose, sodium pyruvate, and L-glutamine; Cat# 10-013-CV, Corning, NY, USA), serum-free with 1% penicillin–streptomycin (Pen/Strep) solution (ATCC, Cat# 30-2300, Manassas, VA, USA) for a final concentration of 100 U/mL penicillin and 100 ug/mL streptomycin, 1% antimitotic/antimycotic (anti-anti 100x, A5955, Millipore, Millipore Sigma, Burlington, MA, USA), and 10 µM ROCK inhibitor (Y-27632, StemCell Technologies, Cat# 72302, Vancouver, BC, Canada).

### 2.2. Tissue Processing and Dissociation

NSCLC and normal lung tissues were subjected to mechanical dissociation using scalpels to finely mince the samples. As the pieces become smaller, gentle pipetting with a P1000 facilitates the liberation of cell clusters suitable for direct plating. These liberated cells should be collected, centrifuged, and plated. These liberated cells were centrifuged at 300× *g* for 10 min, the supernatant removed, and the pellet washed twice with HBSS before plating into an ultra-low-attachment dish containing LCIC-supplemented media. To remove immune cells, an additional centrifugation step at 200× *g* for 3 min in 50 mL HBSS was performed, with immune cells remaining in the supernatant. If dissociation was incomplete, the second step was initiated. Remaining chunks of minced tissue were then incubated in 1× triple enzyme digestion mix (prepared from 10× stock containing 1 g Collagenase, 20,000 units DNase, and 100 mg Hyaluronidase in 100 mL HBSS) supplemented with 10 µM ROCK inhibitor. Samples were shaken at room temperature in 30 min intervals, with periodic assessment for adequate digestion. Liberated single cells were either plated for culture, frozen for later use, or subjected to further processing as needed. CD3 microbeads were used to isolate tumor-infiltrating lymphocytes (TILs), which were then frozen. CD45 microbeads were used to remove other immune cells, which were also frozen following the microbead protocol. Primary lung fibroblasts were isolated by plating cells onto tissue culture-treated flasks in DMEM 10% FBS and confirmed by staining with PDGFRa and PDGFRb. Cell viability was assessed using flow cytometry with PI. To confirm the tumor origin of the sample, FACS analysis was performed using antibodies targeting EpCAM.

### 2.3. Cell Culture and Co-Culture Assays

We first isolated cancer cells by sorting dissociated tissue for EpCAM+ cells via flow cytometry and expanding NSCLC cell numbers for no more than a week in specialized cancer-initiating cell medium ([15]). Normal lung epithelial cells were grown in lung cancer medium supplemented with Wnt3a and Noggin ([16]). MHC class I expression was assessed by flow cytometry (HLA-ABC antibody). Cancer cells derived from five patient-derived NSCLC specimens were cultured in cancer-initiating cell medium and treated with various agents to enhance neoantigen expression and accumulation, suppress tolerogenic pathways, and improve immunogenicity. Treatments included 40 ng/mL EGF, 20 ng/mL bFGF, 5 µM A83-01, 25 µM Z-VAD-FMK, 500 nM decitabine, and 1 µg/mL atezolizumab (added at the time of co-culture). Autologous peripheral blood lymphocytes were isolated and subjected to ex vivo expansion in TexMACS medium supplemented with IL-2. We investigated whether we generated activated T cells through our procedure by performing lymphocyte proliferation assays and interferon-gamma ELISAs.

### 2.4. Whole-Exome and Bulk mRNA Sequencing

DNA and RNA were extracted from freshly collected primary tumor and matched adjacent normal tissues using the QIAGEN Quick-DNA and Quick-RNA Microprep Kits, respectively. Whole-exome sequencing (WES) was performed on tumor and matched normal DNA using the Agilent SureSelect Human All Exon V7 kit for library preparation, followed by 150 bp paired-end sequencing on the Illumina NovaSeq 6000 platform. Raw reads were aligned to the human reference genome (GRCh38) using BWA-MEM, and somatic variants were identified using GATK Mutect2. Bulk RNA sequencing (RNA-seq) was conducted on tumor tissue using the Clontech SMARTer Stranded Total RNA-Seq Pico Input Mammalian v3 Kit and sequenced on the same platform. RNA-seq reads were aligned to GRCh38 using STAR, and gene expression levels were quantified with featureCounts. Expression data were used to filter for expressed somatic mutations prior to downstream neoantigen prediction.

### 2.5. Neoantigen Prediction Pipeline

To identify neoantigen candidates, we utilized the nextNEOpi pipeline to process raw DNA and RNA sequencing data from paired tumor-normal samples, including those subjected to ImmuniT priming [14]. nextNEOpi is a Nextflow-based computational framework that integrates multiple stages, including data preprocessing, variant identification, HLA typing, neoantigen prediction, and quantification of tumor immunogenicity-related features. For class-I neoepitope prediction, nextNEOpi employs pVACseq, which incorporates netMHCpan, netMHCIIpan, and mhcflurry, all of which leverage machine-learning algorithms to predict MHC-presented neoantigens. Fusion neoepitopes were identified using NeoFuse. Raw whole-exome sequencing (WES) and RNA sequencing data underwent quality control to remove low-quality reads, adapter sequences, and contaminants. Somatic variants were identified by comparing tumor and normal WES data, and HLA typing was performed to determine the MHC molecules expressed by tumor cells. Neoantigen candidates were then predicted based on somatic variant-derived peptides capable of MHC class I presentation on the tumor cell surface. Predictions were confirmed against the TESLA data results outlined in [13].

### 2.6. Neoantigen Expression Assays

Lentiviral vectors encoding full-length peptide sequences of predicted neoantigens were designed and constructed by VectorBuilder. To generate lentiviruses, HEK293T cells were transfected with the lentivector and packaging plasmids using Lipofectamine 2000, following the manufacturer’s protocol (Thermo Fisher Scientific, Waltham, MA, USA). Viral supernatants were collected at 24 and 48 h post-transfection and concentrated by incubating with a 50% PEG solution in sterile PBS for 24 h. The resulting viral precipitate was pelleted by centrifugation at 2500 rpm for 20 min, after which the supernatant was discarded, and the viral pellet was resuspended in PBS. Viral titers were determined using a P24 ELISA kit. For transduction, primary patient-derived lung fibroblasts at 50% confluence were exposed to the concentrated lentivirus at a multiplicity of infection (MOI) of 1 in the presence of 8 µg/mL polybrene.

### 2.7. Tetramer Staining and Flow Cytometry

Custom MHC–peptide tetramers were designed and produced by BioLegend to detect antigen-specific T cells in non-small cell lung cancer (NSCLC) patient samples. The tetramers used in this study included HLA-A*24:02, MED23 (TYSRLLVCM-PE) and HLA-A*03:01, SNTB2 (ATSTAGCSK-PE). Peripheral blood mononuclear cells (PBMCs) or tumor-infiltrating lymphocytes (TILs) were first enriched for T cells using CD3 MicroBeads (Miltenyi Biotec) and magnetic-activated cell sorting (MACS) to isolate CD3^+^ T cells. The purified cells were resuspended in staining buffer (PBS + 2% FBS) at $1\times 10^{6}$ cells per 100 µL and incubated with PE-labeled tetramers at room temperature for 30 min in the dark. Flow cytometry analysis was performed on a BD Fortessa high-parameter flow cytometer, and data were analyzed using FCS Express software (FCS Express 7). Tetramer-positive T cells were identified by gating on live, single cells, with proper fluorescence compensation and gating strategies applied using fluorescence-minus-one (FMO) controls and unstained samples.

### 2.8. In Silico Neoantigen Benchmarking

Known tumor-associated antigens (TAAs), including NY-ESO-1 and CEA (CAP1-6D analog), were selected based on prior studies demonstrating expression in NSCLC and immunogenicity. Peptides with reported HLA-restriction and immunogenicity were prioritized. Peptide-HLA binding affinities were evaluated using NetMHCpan 4.1 and IEDB’s MHC I Binding Prediction Tool (accessed May–July 2025). Binding affinities were expressed in terms of IC50 values (nM) and percentile ranks, with strong binders defined as IC50 < 50 nM and/or percentile rank < 0.5%. Comparisons were made with native and analog peptides (e.g., NY-ESO-1 SLLMWITQA vs. SLLMWITQC). Each peptide was cross-validated with the Immune Epitope Database (IEDB) to confirm documented immunogenicity and use in T cell assays or clinical trials. PubMed and ClinicalTrials.gov were queried to assess relevance to NSCLC or other carcinomas. Only peptides with experimental support for MHC presentation and T cell activation were included.

### 2.9. Statistical Analysis

Each experimental group consisted of three independent biological replicates, with each experiment performed in technical triplicates. Statistical analyses were conducted using GraphPad Prism v10. For all comparisons involving four groups (ImmuniT, standard, unstim, and CD3/CD28), one-way ANOVA was performed followed by Tukey’s Honest Significant Difference (HSD) post hoc test to control for multiple comparisons. Normality and homogeneity of variances were assessed using the Shapiro–Wilk and Levene’s tests, respectively. Effect sizes and 95% confidence intervals were calculated where applicable. A *p*-value < 0.05 was considered statistically significant. For in silico analyses, the nextNEOpi pipeline was used, incorporating NetMHCpan and related machine learning models to prioritize neoantigens based on binding rank and IC50 affinity thresholds.

## 3. Results

### 3.1. Patient Characteristics and Tumor Stage

**Figure 1:**
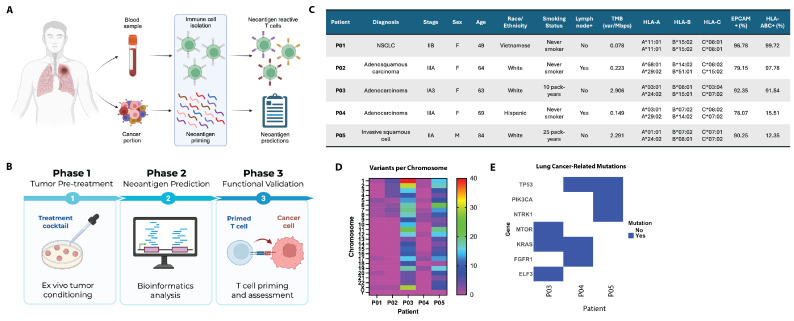
Overview of the ImmuniT platform and patient characteristics. (**A**) Schematic of the ImmuniT platform workflow, which integrates immune cell and tumor isolation from patient samples to enable neoantigen prediction and priming of reactive T cells. (**B**) The platform consists of three phases: Phase 1 involves ex vivo tumor pre-treatment using a defined cocktail to enhance antigen presentation; Phase 2 uses bioinformatics tools for neoantigen prediction; and Phase 3 entails performing a functional validation of T cell priming and cytotoxicity against treated tumor cells. (**C**) Clinical and molecular characteristics of five lung cancer patients analyzed in this study, including diagnosis, smoking status, tumor mutational burden (TMB), HLA typing, and surface marker expression. (**D**) Heatmap showing the number of genetic variants per chromosome across patients P01–P05. (**E**) Bar plot highlighting the presence of key lung cancer-associated gene mutations in patients P03, P04, and P05.

To demonstrate the applicability of the ImmuniT platform for personalized neoantigen discovery and validation, we processed tumor and matched blood samples from five lung cancer patients with varying histological subtypes, clinical features, and tumor mutational burdens (Figure 1). Patients who consented to donate tissue and blood were enrolled in this IRB-approved study. Freshly collected lung cancer tissue from resected specimens, in excess of clinical need along with matched blood samples, were processed into single-cell suspensions (Figure 1A). The ImmuniT platform was implemented in three phases: (1) ex vivo tumor pre-conditioning using a defined treatment cocktail to enhance antigen expression, (2) computational prediction of tumor-specific neoantigens using integrated genomic and transcriptomic data, and (3) functional validation of neoantigen immunogenicity through T cell priming and assessment (Figure 1B). To enhance neoantigen detection, cancer cells were treated to amplify antigen expression using a cocktail of drugs that variously activate protein synthesis, block TGF-β signaling, block caspase activity, promote DNA demethylation, and suppress tolerogenic pathways. Tumor conditioning in Phase 1 improved antigen expression, thus improving the sensitivity of subsequent detection. In Phase 2, bioinformatics tools were applied to generate individualized neoantigen predictions for each patient. Additionally, non-primed T cells were incorporated into the platform to directly enrich for neoantigen-reactive T cells post-treatment with the ImmuniT platform. Figure 1C presents clinical and molecular data for five NSCLC patients, demonstrating substantial variability in cancer subtype, tumor stage, and molecular characteristics. These cases included non-small cell lung cancer (NSCLC), adenocarcinoma, adenosquamous carcinoma, and invasive squamous cell carcinoma. Among the five patients, four are female and one is male, with ages ranging from 49 to 84 years and racial/ethnic backgrounds including Vietnamese, White, and Hispanic individuals. Smoking history varies, with three patients identified as never-smokers, while two (patients P03 and P05) have smoking histories of 10 and 25 pack-years, respectively. Disease stage ranges from IA3 to IIIA, indicating varying tumor progression with two patients (P02 and P04) exhibiting lymph node metastasis.

HLA genotyping reveals distinct allele combinations for HLA-A, HLA-B, and HLA-C across patients, which can influence antigen presentation and immune recognition. Additionally, expression levels of EpCAM and HLA-ABC highlight notable molecular differences. EpCAM positivity ranges from 78.07% to 96.78%, while HLA-ABC expression is highly variable, with patients P05 and P04 exhibiting only 12.35% and 15.51% positivity, respectively, in contrast to the high expression observed in patients P01 (99.72%), P02 (97.78%), and P03 (91.54%) (Figure 1C). HLA expression and its complexing with neoepitopes are essential for TCR-mediated recognition and tumor cell elimination [17]. Consequently, low HLA expression facilitates immune evasion and diminishes the effectiveness of immunotherapy. Tumor mutational burden (TMB), measured in variants per megabase, spans from 0.078 to 2.906 across patients. As shown in Figure 1C, patients P03 and P05, both former smokers, exhibit higher TMB compared to non-smokers, reinforcing the established link between smoking history and increased mutational load [18]. However, even among former smokers, TMB remained below the NSCLC median of 9.8 mutations/Mb [19], suggesting significant variability in mutation-driven antigenicity. Variant profiling across the genome revealed patient-specific mutation distributions, with the highest frequency of variants detected on chromosomes 1, 3, and 17 (Figure 1D). The heatmap also highlights differences between patients in the distribution of mutations across chromosomes, with former smokers displaying a broader range of mutations, indicative of greater genomic instability (Figure 1D).

Figure 1E further provides insights into lung cancer-related mutations, revealing differences in oncogenic alterations between patients. P04 and P05 harbor mutations in TP53, a key tumor-suppressor gene, while P03 and P04 carry mutations in KRAS, a common driver in lung cancer [20]. P03 also has mutations in MTOR and ELF3, while P04 has mutations in FGFR1, and P05 has mutations in PIK3CA and NTRK1–genes involved in tumor growth and survival [21]. Interestingly, patients P01 and P02 lack mutations in these well-known oncogenic drivers, with an alternative mutational landscape contributing to tumor progression. These findings highlight the genetic heterogeneity of NSCLC, emphasizing the need for personalized therapeutic approaches based on individual mutation profiles.

### 3.2. T Cell Activation and Neoantigen Prediction with the ImmuniT Platform

**Figure 2:**
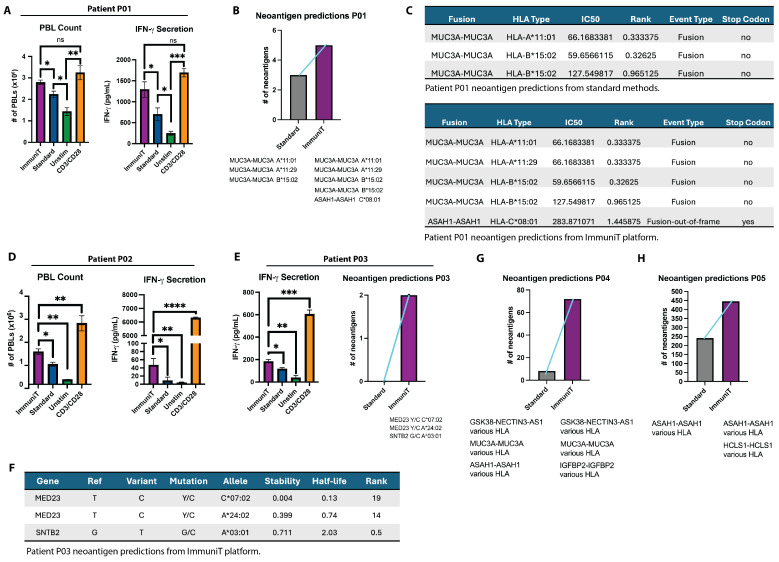
ImmuniT platform shows improved T cell activation and neoantigen detection compared to standard methods. (**A**) Quantification of peripheral blood lymphocyte (PBL) proliferation and IFN-γ secretion from patient P01 under unstimulated, standard priming, CD3/CD28 stimulation, and ImmuniT conditions. One-way ANOVA revealed significant differences (*p* = 4.67 × 10^−6^), with Tukey’s post hoc test confirming that the ImmuniT group induced significantly higher responses compared to standard and unstimulated conditions (*p* < 0.001). (**B**) Plot shows that standard methods detected three neoantigens for patient P01 lung cancer specimen, whereas ImmuniT platform detected five neoantigens. Neoantigens are indicated below column. (**C**) Table showing characteristics of fusion neoantigens detected by standard methods and ImmuniT platform for patient P01 lung cancer. (**D**) Peripheral blood lymphocyte (PBL) proliferation from patient P02 following stimulation with standard, ImmuniT-treated tumor cells, unstimulated controls, and CD3/CD28 stimulation. ImmuniT treatment significantly increased T cell proliferation (*p* = 2.86 × 10^−5^, one-way ANOVA; *p* < 0.001, Tukey HSD post hoc test). (**E**) IFN-γ secretion from patient P03 PBLs following co-culture with standard, ImmuniT-treated tumor cells, unstimulated controls, and CD3/CD28 stimulation. One-way ANOVA identified significant differences among conditions (*p* = 1.70 × 10^−7^), with Tukey HSD post hoc tests confirming significantly greater IFN-γ secretion in the ImmuniT group compared to other conditions (*p* < 0.0001). Plot shows that standard methods did not detect any neoantigens for patient P03 lung cancer specimen, whereas ImmuniT platform detected 2 neoantigens. Neoantigens are indicated below column. (**F**) Table, showing the characteristics of neoantigens detected by ImmuniT platform for patient P03 lung cancer. (**G**) Plot shows that standard methods detected 10 neoantigens for patient P04 lung cancer specimen, whereas ImmuniT platform detected 70 neoantigens. Neoantigen classes are indicated below columns. (**H**) Plot shows that standard methods detected 225 neoantigens for patient P05 lung cancer cells, whereas ImmuniT platform detected 450 neoantigens. Neoantigens are indicated below columns. * *p*-value < 0.05, ** *p*-value < 0.01, *** *p*-value < 0.001, **** *p*-value < 0.0001.

To evaluate the ability of the ImmuniT platform to improve T cell priming and activation compared to standard methods, we assessed effects of tumor cell pre-treatment on the activation of patient-derived T cells from individuals with high MHC class I expression on the tumor (HLA-ABC, assessed via flow cytometry). For patient P01, tumor pre-treatment with the ImmuniT platform (Phase 1) significantly enhanced subsequent T cell proliferation and activation (Phase 3), surpassing the response observed in non-treated (standard) tumor-primed T cells (Figure 2A; one-way ANOVA *p* = 4.67 × 10^−6^; Tukey HSD *p* < 0.001 for ImmuniT vs. STD and UNSTIM). In contrast, unstimulated peripheral blood lymphocytes (PBLs) exhibited minimal proliferation, while CD3/CD28 agonist (TRANSACT)-stimulated T cells showed nonspecific activation. Notably, IFN-γ secretion was markedly elevated in T cells exposed to ImmuniT-treated cancer cells, confirming robust immune activation and highlighting the platform’s potential to enhance immune responses.

To determine whether the ImmuniT platform improves neoantigen detection (Phase 2), we integrated next-generation sequencing (NGS) and a bioinformatics pipeline to analyze primary NSCLC tumors from patients P01, P03, P04, and P05. For patient P01, standard approaches to tumor cell handling and neoantigen prediction yielded a limited set of three gene fusion–derived neoantigens (MUC3A-MUC3A epitopes, presented by various HLA types with differing stability and affinity) (Figure 2B,C). In contrast, the ImmuniT platform identified two additional fusion neoantigens: an alternative MUC3A fusion and an ASAH1-ASAH1 out-of-frame fusion (Figure 2B). Features of the predicted neoantigens, including IC50 values and HLA binding ranks, are presented in Figure 2C. The IC50 value denotes the concentration of peptide (in nanomolars) required to inhibit 50% the binding of a reference peptide to an MHC molecule, and serves as a surrogate for the peptide–MHC binding affinity. NetMHCpan uses both IC50 and the percent rank to assign a binding level label, with lower IC50 values corresponding to stronger predicted binding. Similarly, the HLA binding rank provides a percentile-based comparison across alleles, where lower ranks indicate a higher predicted affinity. These values were computed using NetMHCpan and used to prioritize candidate neoantigens with the highest potential for effective MHC presentation and immunogenicity.

A similar increase in T cell activation was observed in patient P02, where ImmuniT pre-treatment led to a significant increase in T cell proliferation compared to standard priming (Figure 2D; one-way ANOVA *p* = 2.86 × 10^−5^; Tukey HSD *p* = 0.0004 for ImmuniT vs. STD and *p* = 0.0001 vs. UNSTIM), further demonstrating the ability of the platform to improve immune responsiveness. Similarly, Patient P03 exhibited a notable increase in IFN-γ secretion following ImmuniT treatment (Figure 2E; one-way ANOVA *p* = 1.70 × 10^−7^; Tukey HSD *p* < 0.0001 for ImmuniT vs. STD and UNSTIM). For patient P03, the ImmuniT platform also uncovered two novel neoantigens, MED23 and SNTB2, both implicated in lung cancer progression [22,23]. These neoantigens were undetectable using standard workflows to assess neoantigen expression in cancer cells, underscoring ImmuniT’s ability to reveal tumor antigens with potential therapeutic relevance. Characterization confirmed HLA specificity, stability, and functional properties, supporting their potential for immunotherapy (Figure 2F).

Using the ImmuniT platform, 70 fusion-derived neoantigens were identified in patient P04, including GSK3B–NECTIN3-AS1, MUC3A–MUC3A, and ASAH1–ASAH1, compared to only 10 detected using standard methods. Furthermore, IGFBP2-IGFBP2 emerged as a unique ImmuniT-identified target (Figure 2G). IGFBP2 (Insulin-like Growth Factor Binding Protein 2) acts as an oncogene, promoting tumor growth, migration, and invasion, and it can serve as a potential therapeutic target and biomarker [24]. These fusion neoantigens represent tumor-specific junctional peptides arising from gene fusions or intragenic rearrangements, and are particularly compelling immunotherapy targets due to their unique, tumor-restricted sequences and strong potential for immunogenicity. In patient P05, 225 neoantigens (ASAH1-ASAH1, various HLA types) were predicted by standard methods, whereas 450 neoantigens (ASAH1-ASAH1 and HCLS1-HCLS1, various HLA types) were identified using ImmuniT (Figure 2H, Supplemental Tables). The ASAH1 gene, which encodes the enzyme acid ceramidase, has been implicated in cancer progression [25]. Collectively, these findings suggest that the ImmuniT platform enhances antigen-specific detection and T cell priming, a critical factor for effective cancer immunotherapy.

### 3.3. Functional Validation of Novel Neoantigens

**Figure 3:**
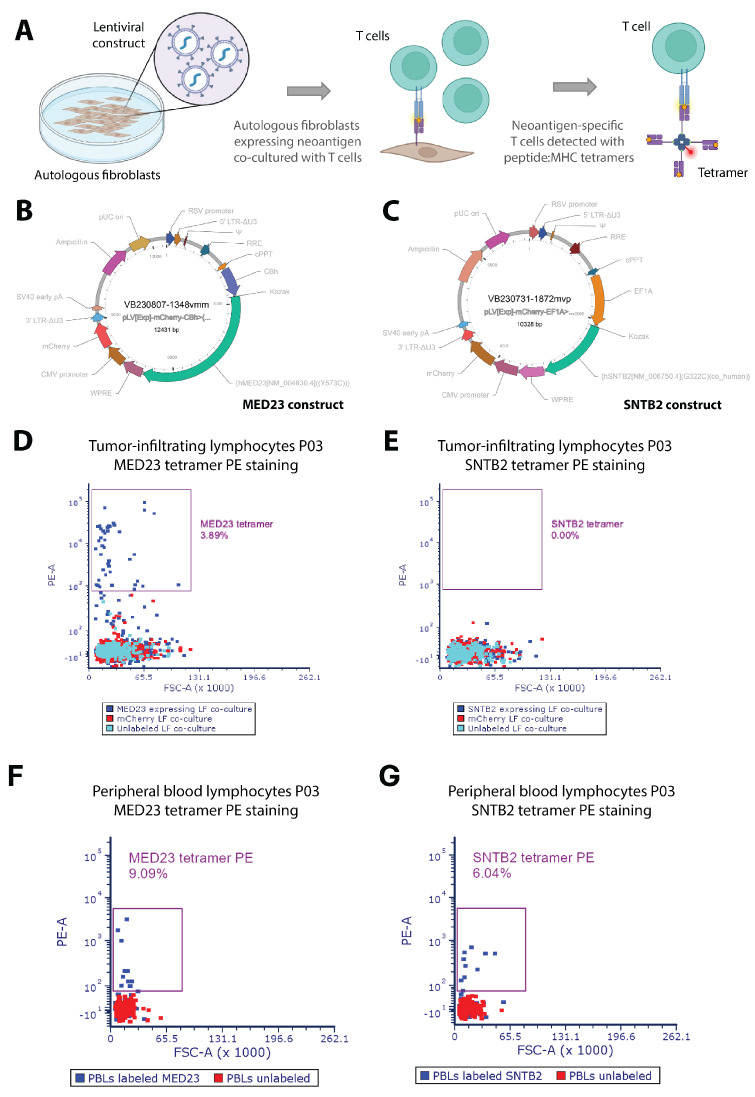
The ImmuniT platform enhances the identification of neoantigens for patient P03, as shown by the expansion of neoantigen-specific tumor-infiltrating lymphocytes detected through peptide:MHC tetramers. (**A**) Schematic showing workflow for assessing TCR specificity. Autologous fibroblasts are transduced with a lentiviral construct containing the neoantigen of interest, then co-cultured with patient-specific T cells. T cells with TCR specific to neoepitope MHC present on the cell surface are enriched and detected via peptide–MHC tetramer staining and flow cytometry. (**B**) Plasmid containing a neoantigen construct and fluorescent reporter for MED23 and (**C**) SNTB2; putative neoantigens detected by ImmuniT platform for patient P03. (**D**) Flow cytometric analyses of tumor-infiltrating lymphocytes (TILs) from patient P03 co-cultured with MED23 expressing autologous lung fibroblasts (LF), in addition to TILs co-cultured with control mCherry expressing LF and unlabeled LF for MED23 and (**E**) SNTB2 peptide–MHC tetramer staining. Note that 3.89% of TILs co-cultured with MED23 expressing LF show TCR specificity for MED23, whereas 0% of TILs co-cultured with SNTB2 expressing LF show TCR specificity for SNTB2. (**F**) Flow cytometric analyses of peripheral blood lymphocytes (PBLs) from patient P03 co-cultured with MED23 expressing autologous lung fibroblasts (LF) in addition to PBLs with unlabeled LF for MED23 and (**G**) SNTB2 peptide–MHC tetramer staining. Note that 9.09% of PBLs show TCR specificity for MED23, whereas 6.04% of PBLs show TCR specificity for SNTB2.

To validate the functional avidity of T cells from patient P03, who had a high mutational burden and high expression of HLA-ABC, against the predicted neoantigens, we employed a cloning and expression system to investigate T cell receptor (TCR) specificity in TILs from patient P03 (Figure 3A). Custom-designed oligonucleotides encoding the full-length neoantigen peptide sequences for MED23 and SNTB2 were cloned into a lentiviral plasmid and used to produce lentiviral particles, enabling the stable transduction of autologous fibroblasts for neoantigen overexpression (Figure 3B,C). Transduction efficiency was confirmed via fluorescence markers, ensuring robust antigen presentation. Co-culture experiments with neoantigen-expressing fibroblasts and patient-derived TILs revealed a 3.84% frequency of MED23-specific T cells, as confirmed by flow cytometry and peptide–MHC (pMHC) tetramer staining (Figure 3D). No SNTB2-specific TILs were detected (Figure 3E). However, analysis of peripheral blood lymphocytes (PBLs) demonstrated the presence of T cells reactive to both neoantigens (Figure 3F,G), suggesting that neoantigen-specific T cell populations may differ between peripheral blood and the tumor microenvironment. This observation could have significant implications for neoantigen-targeted immunotherapy, as circulating T cells might serve as a reservoir for antigen-specific immune responses.

To contextualize the predicted immunogenicity of the two patient-specific neoantigens, MED23 (Y573C) and SNTB2 (G322C), we performed a comparative benchmarking analysis against a curated panel of well-characterized antigens relevant to NSCLC. This reference panel included shared cancer/testis antigens (MAGE-A1 [26], MAGE-A3 [27,28], NY-ESO-1 [27,29]), lineage-associated antigens (CEA [CAP1-6D analog] [30,31], MUC1 [32,33]), tumor-associated self-antigens (Survivin [31], hTERT [31]), and neoepitopes derived from recurrent driver mutations (KRAS G12C/D/V [34,35] and TP53 K132N [34]). As shown in Table 1, both MED23 and SNTB2 displayed strong predicted MHC class I binding affinity, with IC50 values of 332.17 nM (HLA-A*24:02) and 183.51 nM (HLA-A*03:01), respectively, placing them in the strong binder category based on NetMHCpan criteria. Their binding ranks (0.4258 and 0.599, respectively) further support their capacity to engage HLA molecules effectively. Although their predicted immunogenicity scores were modest (−0.0195 for MED23 and −0.0868 for SNTB2), these values are comparable to those observed for several benchmark tumor-associated antigens, including CEA, hTERT, and TP53 (K132N). This suggests that despite their novel and patient-specific origin, both neoantigens exhibit a predicted T cell recognition profile on par with clinically investigated antigens, highlighting their potential relevance for immunotherapeutic targeting in NSCLC.

## 4. Discussion

Our study reveals that conventional neoantigen identification methods may miss functionally relevant targets, potentially limiting the efficacy of personalized cancer vaccines and adoptive T cell therapies. Using the ImmuniT platform, we achieved enhanced T cell activation and proliferation, improved neoantigen detection, and functional validation of neoantigen-specific T cells, uncovering the differential distribution of neoantigen-specific T cells between peripheral blood-circulating lymphocyte populations and tumor resident compartments. In our functional analysis of patient P03, flow cytometry and pMHC tetramer assays demonstrated that while tumor-infiltrating lymphocytes (TILs) contained MED23-specific T cells, no SNTB2-specific T cells were detectable within the tumor microenvironment. In contrast, MED23- and SNTB2-reactive T cells were both present in peripheral blood lymphocytes (PBLs). This observed compartmental bias suggests that peripheral blood may represent a more accessible reservoir of neoantigen-specific T cells, potentially reflecting immune exclusion mechanisms within the tumor microenvironment. However, it also raises the possibility that technical artifacts introduced during T-cell isolation, in vitro culture, or expansion protocols may influence the apparent distribution.

These findings have significant implications for both vaccine-based and cell-based immunotherapies. For cell-based therapies, selecting the optimal starting population is crucial for approaches such as TIL therapy or the adoptive transfer of neoantigen-specific T cells [8,11,36,37]. The underrepresentation or functional suppression of certain tumor-reactive clones within the tumor microenvironment may limit therapeutic efficacy, and current expansion protocols might preferentially amplify T cells with superior growth characteristics rather than those with the highest tumor reactivity [38,39]. For vaccine-based therapies, therapeutic vaccines depend on the priming and expansion of endogenous neoantigen-reactive T cells to generate a robust anti-tumor response [9,40]. By enhancing neoantigen identification, the ImmuniT platform could improve vaccine efficacy by expanding rare but functionally significant T cell clones before antigen exposure. Given the observed biases in the distribution of neoantigen-specific T cells, understanding the compartmental distribution of neoantigen-specific T cells is therefore essential to optimizing immunotherapy and improving patient outcomes. Additionally, the selective pressures within the tumor microenvironment may deplete highly functional T cell clones, further complicating efforts to harness an effective anti-tumor response.

The functional relevance of predicted neoantigens remains a key challenge in immunotherapy development. In the absence of a tetramer-based positive control, we benchmarked our novel antigens against a panel of established tumor antigens associated with NSCLC. This comparative analysis revealed that MED23 (Y573C) and SNTB2 (G322C) possess MHC binding and immunogenicity scores consistent with several validated and clinically explored antigens, including MAGE-A1, NY-ESO-1, and TP53 K132N. Importantly, while immunogenicity prediction scores can underpredict the functional potential of rare or unconventional epitopes such as those derived from tumor-specific mutations and alone do not fully capture antigen processing, presentation, or T cell repertoire constraints, they offer a useful comparative framework when placed alongside widely studied NSCLC antigens. Our findings support the notion that MED23 and SNTB2 are viable candidates for further experimental validation and reinforce the importance of incorporating shared neoantigen comparisons in early-stage validation efforts.

While this study serves as a proof of concept, a key limitation is the small sample size (n = 5 patients), which restricts the generalizability of the findings and precludes robust subgroup analyses. Furthermore, the patient cohort was heterogeneous with respect to factors known to influence immune responses, including tumor type, disease stage, smoking history, and HLA genotype. Although such diversity mirrors real-world clinical complexity, it complicates interpretation and underscores the need for validation in larger, stratified cohorts. Future studies should investigate whether the ImmuniT platform can be broadly applied across clinical subtypes or whether tailored approaches are required. Despite these limitations, the consistent enhancement of neoantigen-specific T cell responses in all five patients supports the translational promise of this platform.

By integrating next-generation sequencing with functional validation assays, our study demonstrates that the ImmuniT platform can identify rare but immunologically significant neoantigens, even in NSCLC tumors with low tumor mutational burden. The platform improves both the sensitivity and breadth of neoantigen detection, uncovering epitopes that are overlooked by conventional approaches. Another key strength of the platform is its HLA-agnostic approach, which enables the identification and validation of neoantigens without being constrained by HLA restrictions. Moving forward, efforts should prioritize refining neoantigen-specific T cell expansion protocols and optimizing therapy designs to address compartmental biases and functional constraints. By enhancing T cell activation, improving neoantigen detection, and circumventing the challenges associated with low TMB, the ImmuniT platform establishes a powerful foundation for developing highly personalized and broadly accessible immunotherapies.

## 5. Conclusions

Our findings underscore the limitations of conventional neoantigen discovery pipelines and highlight the value of integrating functional validation into neoantigen identification workflows. The ImmuniT platform offers a robust, HLA-agnostic solution to uncovering immunologically meaningful neoantigens, even in tumors with low mutational burden. By revealing compartment-specific biases in neoantigen-specific T cell distribution and demonstrating enhanced T cell activation and expansion using the ImmuniT platform, this study emphasizes the need to reassess both the sources and functional states of T cells used in immunotherapy. These findings, while based on a small and heterogeneous patient cohort, demonstrate the feasibility of using an integrated in vitro and in silico strategy for neoantigen discovery. Future studies in larger, stratified cohorts will be critical to assess the broader applicability of the ImmuniT platform across diverse clinical profiles. Ultimately, the ImmuniT platform may advance the precision of both vaccine-based and cell-based strategies, paving the way for more effective personalized cancer immunotherapies that overcome current limitations in neoantigen targeting.

## Figures and Tables

**Table 1:** Benchmarking of MED23 and SNTB2 neoantigens against validated tumor-associated and recurrent neoantigens. Binding ranks and affinities were predicted using NetMHCpan 4.1. Immunogenicity scores were determined using the IEDB immunogenicity prediction tool.

Antigen Name	Peptide Sequence	IEDB ID	HLA Allele	Affinity Rank	Affinity Score	Immunogenicity	Affinity (nM)	Binding Level
CEA	YLSGANLNL	74915	A*02:01	0.105	0.7986	−0.00073	8.84	Strong
KRAS (G12C)	VVGACGVGK	–	A*11:01	0.427	0.6035	0.08441	72.99	Weak
KRAS (G12D)	VVGADGVGK	–	A*03:01	0.286	0.6045	0.24736	72.17	Strong
KRAS (G12V)	VVGAVGVGK	–	A*11:01	0.384	0.6140	0.17711	65.10	Strong
MAGE-A1	KVLEYVIKV	34095	A*02:01	0.054	0.8371	0.11873	5.83	Strong
MAGE-A3	FLWGPRALV	16970	A*02:01	0.083	0.8120	0.17046	7.65	Strong
MED23 (Y573C)	TYSRLLVCM	–	A*24:02	0.426	0.0002	−0.01952	332.17	Strong
MUC1	LLLLTVLTV	37528	A*02:01	0.289	0.7231	0.07520	20.01	Strong
NY-ESO-1	SLLMWITQC	59278	A*02:01	2.553	0.4413	0.12576	422.15	Weak
SNTB2 (G322C)	ATSTAGCSK	–	A*03:01	0.599	0.5183	−0.08680	183.51	Strong
Survivin	LTLGEFLKL	237188	A*02:01	4.719	4.7190	0.10284	1344.98	Weak
hTERT	YLFFYRKSV	2255432	A*02:01	0.180	0.7606	−0.07774	13.34	Strong
TP53 (K132N)	TYSPALNNM	–	C*07:02	0.066	0.5441	−0.04644	138.82	Strong

## Data Availability

The original data presented in the study are openly available in Gene Expression Omnibus (GEO) at [GSE306693].

## Supplementary Materials

The following supporting information can be downloaded at: https://www.mdpi.com/article/10.3390/vaccines13090921/s1, Table S1: Neoantigen predictions for individual patients with and without the ImmuniT platform.
